# PW03-033 - SLC29A3 mutation: a new autoinflammatory condition

**DOI:** 10.1186/1546-0096-11-S1-A259

**Published:** 2013-11-08

**Authors:** I Melki, K Lambot, L Jonard, V Couloigner, P Quartier, B Neven, B Bader-Meunier

**Affiliations:** 1 Unité d'Immunologie Hématologie et Rhumatologie Pédiatrique, Necker-Enfants Malades Hospital, Paris, France; 2 Service de Pédiatrie Générale, Robert Debré Hospital, France; 3 Department of pediatric radiology, Necker-Enfants Malades Hospital, France; 4 Department of genetics, Trouseau Hospital, France; 5 Institut IMAGINE, Paris Descartes University, Sorbonne Paris Cité, France; 6 Department of Microbiology, Necker Enfants Malades Hospital, France; 7 U768, INSERM, Paris, France

## Introduction

Germline mutations in *SLC29A3* result in a range of clinically related, recessive syndromes: H syndrome, pigmented hypertrichosis with insulin-dependent diabetes mellitus (PHID) syndrome, Faisalabad histiocytosis (FHC), and sinus histiocytosis with massive lymphadenopathy (SHML). Main symptoms of these diseases are hyperpigmentation with hypertrichosis, sensorineural deafness, diabetes, short stature, uveitis and “Rosai-Dorfman-like” histiocytosis.

## Case report

We report the case of an eleven-month-old boy with early-onset recurrent episodes of unprovoked fever lasting 7 to 10 days associated with pericardial effusion, abdominal pain, diarrhea, and inflammation. Physical examination revealed hyperpigmentation with hypertrichosis, dysmorphic features and a spleen and liver enlargement. Failure to thrive, sensorineural deafness, psychomotor development delay, and a “Rosai-Dorfman like” cheek lesion further developed. Febrile attacks were not responsive to interleukin-1 and Tumor-Necrosis-Factor blocking agents. All known causes of genetic autoinflammatory syndromes were excluded by sequencing (*MEFV*, *NALP3*, *mevalonate kinase*, *NALP12*, *TNFRSF1*). Sequencing of *SLC29A3* gene revealed homozygous missense mutation c.1088G>A (p.Arg363Gln).

## Discussion

This case is the first description of a patient with an auto-inflammatory disorder due to a mutation in *SLC29A3* gene. Genetic defect of *SLC29A3* should be considered in patients with recurrent febrile attacks associated with any symptoms reminiscent of *SLC29A3* broad spectrum of manifestations, especially hyperpigmentation with hypertrichosis.

## Disclosure of interest

None declared

